# Novel genetic association of the Furin gene polymorphism rs1981458 with COVID-19 severity among Indian populations

**DOI:** 10.1038/s41598-024-54607-7

**Published:** 2024-04-03

**Authors:** Rudra Kumar Pandey, Anshika Srivastava, Rahul Kumar Mishra, Prajjval Pratap Singh, Gyaneshwer Chaubey

**Affiliations:** Cytogenetics Laboratory, Department of Zoology, Banaras Hindu University, Varanasi, 221005 India

**Keywords:** Genetics, Genetic association study, Haplotypes, Mutation, Population genetics

## Abstract

SARS CoV-2, the causative agent for the ongoing COVID-19 pandemic, it enters the host cell by activating the ACE2 receptor with the help of two proteasesi.e., Furin and TMPRSS2. Therefore, variations in these genes may account for differential susceptibility and severity between populations. Previous studies have shown that the role of ACE2 and TMPRSS2 gene variants in understanding COVID-19 susceptibility among Indian populations. Nevertheless, a knowledge gap exists concerning the COVID-19 susceptibility of Furin gene variants among diverse South Asian ethnic groups. Investigating the role of Furin gene variants and their global phylogeographic structure is essential to comprehensively understanding COVID-19 susceptibility in these populations. We have used 450 samples from diverse Indian states and performed linear regression to analyse the Furin gene variant's with COVID-19 Case Fatality Rate (CFR) that could be epidemiologically associated with disease severity outcomes. Associated genetic variants were further evaluated for their expression and regulatory potential through various Insilco analyses. Additionally, we examined the Furin gene using next-generation sequencing (NGS) data from 393 diverse global samples, with a particular emphasis on South Asia, to investigate its Phylogeographic structure among diverse world populations. We found a significant positive association for the SNP rs1981458 with COVID-19 CFR (p < 0.05) among diverse Indian populations at different timelines of the first and second waves. Further, QTL and other regulatory analyses showed various significant associations for positive regulatory roles of rs1981458 and Furin gene, mainly in Immune cells and virus infection process, highlighting their role in host immunity and viral assembly and processing. The Furin protein–protein interaction suggested that COVID-19 may contribute to Pulmonary arterial hypertension via a typical inflammation mechanism. The phylogeographic architecture of the Furin gene demonstrated a closer genetic affinity of South Asia with West Eurasian populations. Therefore, it is worth proposing that for the Furin gene, the COVID-19 susceptibility of South Asians will be more similar to the West Eurasian population. Our previous studies on the ACE2 and TMPRSS2 genes showed genetic affinity of South Asian with East Eurasians and West Eurasians, respectively. Therefore, with the collective information from these three important genes (ACE2, TMPRSS2 and Furin) we modelled COVID-19 susceptibilityof South Asia in between these two major ancestries with an inclination towards West Eurasia. In conclusion, this study, for the first time, concluded the role of rs1981458 in COVID-19 severity among the Indian population and outlined its regulatory potential.This study also highlights that the genetic structure for COVID-19 susceptibilityof South Asia is distinct, however, inclined to the West Eurasian population. We believe this insight may be utilised as a genetic biomarker to identify vulnerable populations, which might be directly relevant for developing policies and allocating resources more effectively during an epidemic.

## Introduction

COVID-19, an ongoing pandemic is caused by a novel coronavirus SARS-CoV-2. The first case of COVID-19 was observed in the Chinese city of Wuhan in December 2019^1^. Since then, it has claimed the lives of millions and triggered economic turmoil worldwide. The consequences of the COVID-19 catastrophe varies across ethnic groups. Patients from different ethnic origins are disproportionately affected^2^. However, disparities in Cases and case fatality rates (CFR) might be attributed to multiple factors like quarantine and social distancing policies, access to medical care, reliability and coverage of epidemiological data,as well as the Population age structure, which indicates that morbidity is more prevalent in the elderly and comorbidly ill^3,4^. However, acute cytokine storms have also contributed to the deaths of many young and healthy indivisuals^5^. However, It is essential to highlight that these characteristics do not fully explain all the disparities between groups and there are important gaps that require the scientific community's attention to propose and test theories that will assist in understanding the ailment aetiology of COVID-19. However, nations with strict guidelines for gathering and reporting epidemiological data suggest that human genetic variation may be responsible for disease severity and susceptibility differences among various populations^6^.

SARS CoV-2 engages the ACE2 receptor to enter the host cell with the help of two proteases, i.e., Furin and TMPRSS2, which cleave the spike protein of the virus at separate locations (S1/S2 and S2', respectively^7–9^. Variations in these genes may account for differential susceptibility and severity between populations. There is supporting evidence indicating the crucial involvement of ACE2 and TMPRSS2 gene variants in determining COVID-19 susceptibility within Indian populations^10,11^. However, there is a knowledge gap regarding Furin variants among diverse Indian ethnic groups and their impact on disease vulnerability, which needs to be investigated.

Furin is a human protease encoded by the Furin gene^12^. It belongs to the subtilisin-like proprotein convertase enzyme family, which processes and transforms nascent proteins into active forms. A calcium-dependent serine endoprotease, Furin cleaves precursor proteins at their paired basic amino acid processing sites (Arg-X-(Arg/Lys) -Arg' in canonical form). The prevalent presence of Furin in the Golgi body helps proteins attain their active state^13,14^. Various pathogens also employ Furin to cleave the envelope proteins of viruses like HIV, influenza, dengue fever, numerous filoviruses like Ebola, the Marburg virus, and the SARS-CoV-2 spikeprotein^15,16^.

In the context of COVID-19, the Furin protein has been the subject of much discussion, considering its role in activating the SARS-CoV-2 spike protein. Therefore, the effectiveness of this activation pathway may be impacted by the presence of a specific variation in the Furin gene, which may affect the virus ability to infiltrate and infect human cells, in turn modifying the severity of COVID-19 outcomes. Given the importance of the Furin gene in the SARS-CoV-2 infection, variations in this gene could be associated with the disparity in disease susceptibility and severity in various groups. As a result, in this study, we looked for genetic variants of the Furin gene and compared them with the epidemiological data on COVID-19 to test if there is any evidence of an association of this gene variants with the case frequency and case fatality rate among Indian populations. Also, the genetic architecture of the Furin gene is unknown among various world populations. Therefore, we conducted a detailed investigation of the Furin gene sequence data from global populations to identify its Phlogeographic structure among diverse world populations, which may assist in understanding the role of Furin in COVID-19 susceptibility worldwide.

## Material and methods

### Dataset

The frequency of Furin gene variants was obtained using 450 samples from diverse Indian states from Estonian Biocentre^17–19^ data, the 1000 Genomes Project phase 3 data^20^, and our recently genotyped data from various Indian states using Plink 1.9. (21) A total of 4 SNPs (rs4932178, rs17514846, rs1981458, rs4702) were obtained from the genotype data for the Furin gene for which detailed data were available for diverse Indian states and were further studied in detail. The state-wise number of cases and deaths per state from different timelines was collected from https://www.mygov.in/corona-data/covid19-statewise-status/ (Supp. Table S1a,b). State-level frequency maps for rs1981458 and COVID-19 CFR among the Indian population were created using https://www.datawrapper.de/. DbSNP and gnomAD were used to observe the worldwide frequency distribution of rs1981458^22,23^, and its Spatial map was generated using PGG.SNV toolset from 1000 genome data (Table 2 and Supp. Fig. S2)^24^.

### Statistical analysis

To understand how changes in the allele frequency of the Furin gene variant might relate to variations in COVID-19 Case Fatality Rate among different state populations in India, we performed linear regression analysis using 450 samples from diverse Indian states to test the epidemiological association if any, with disease severity. Linear regression attempts to fit a straight line that best represents the relationship between the independent and dependent variables. Linear regression assumes Linearity, Independence, Homoscedasticity, Normality of Residuals and No Multicollinearity of data. SPSS (ver. 26) was used for performing linear regression analysis at a 95% confidence level and 1000 bootstrapping (2,000,000 seeds) for a two-tailed significance (p < 0.05). The composite graphics for all variables were created using an R Studio.

### Phylogeographic analysis

Phylogeographic analysis of the Furin gene across different global populations was carried out using NGS data from Pagani et al.^25^. PLINK 1.9 was employed to retrieve sequences for distinct populations from the dataset^14,21^. We used Principal Component Analysis (PCA) results from Pagani et al. for the quality control, where ethnic outlier samples from Sahul and Africa were removed, along with relatives up to a second degree. A total of 393 samples and 242 SNPs were obtained, which were then utilised in further investigation (Supp. Table S2a,b). A Perl script was used to convert the Plink file to fasta (ped to IUPAC). DNAsp (ver. 6) was used for phasing, Fst computation, Population-wise genetic distances calculation, and Network and Arlequin input file generation^26^. MEGA X was used to construct a Neighbour-joining tree based on Fst^27^. Arlequin 3.5 was used to calculate the average pairwise and Nei's genetic distance, then plotted on the graph using R V3.1^28^. The median-joining (MJ) network was drawn using Network v5 and Network Publisher. Finally, each group's total and prevalent haplotypes in the Furin gene were calculated with an XML file generated with Arlequin 3.5.^28^.

### Insilco analysis

To analyse the FURIN expression in various human tissues, the GTEx portal database (http://www.gtexportal.org/home/) was used.VannoPortal was used for variant annotation and prediction scores from different biological domains^29^. The RegulomeDB database was utilised to annotate SNPs in the intergenic regions of the human genome that correspond to known and predicted regulatory elements^30^. QTLbase was used for different QTLs comparison across multiple tissues to interpret the possible molecular functions of genetic variants and their tissue/cell-type specificity^31^. LDlink, a web-based collection of bioinformatic modules, was used to explore proxy and putatively functional variants for a query variant based on the population groups of interest^32^. An online database and biological resource, STRING (Search Tool for the Retrieval of Interacting Genes/Proteins), was used to identify known and predicted protein–protein interactions queried with FURIN^33^. DICE-Tools was used to compare the Expression of the Furin Gene in Bulk and different Immune cell types; it was also used to analyse gene/protein signatures in relation to Gene ontology (GO) terms and gene expression. Pathway analysis for rs1981458 was done using SNPnexus^34^.

## Results

### Furin gene expression in various tissues

**Figure 1:**
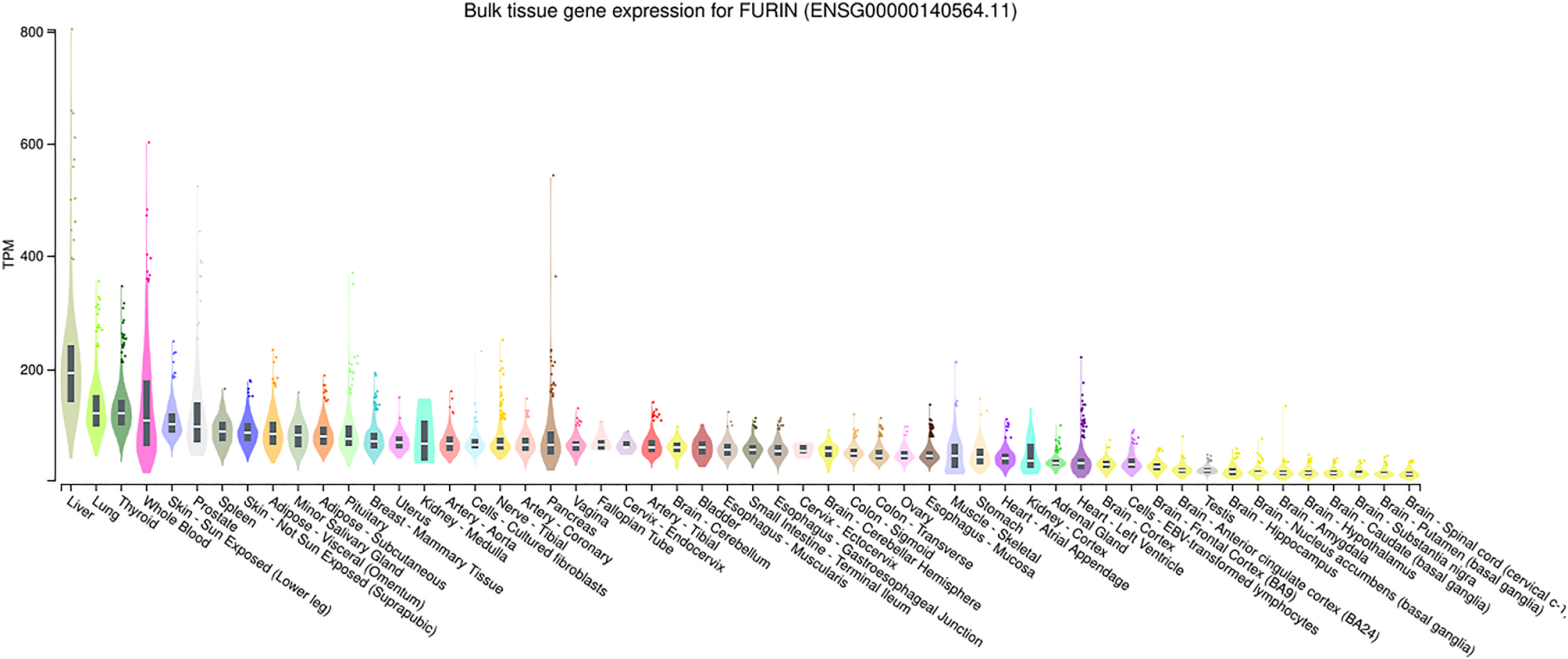
The figure depicts the bulk expression of the Furin gene in various normal tissues sorted based on the ascending order of TPM (Transcription per million value). Box plots are shown as median and 25th and 75th percentile. Points are displayed as outliers above or below 1.5 times the interquartile range.

GTEx database results show that *Furin* has the highest expression in the liver, the lungs, and whole blood while expressed least in brain tissue (Fig. 1). We also looked for Bulk RNA Expression of the Furin Gene in various Immune cell types sorted based on expression score within the cohort. The highest expression was in Nk cells, Monocytes(classical), TG17, TH1 and TREG. Naive B cells and naïve CD4 T cells showed least expression (Supp. Fig. S1 and Supp. Table S3).

### FURIN protein–protein interaction

**Figure 2:**
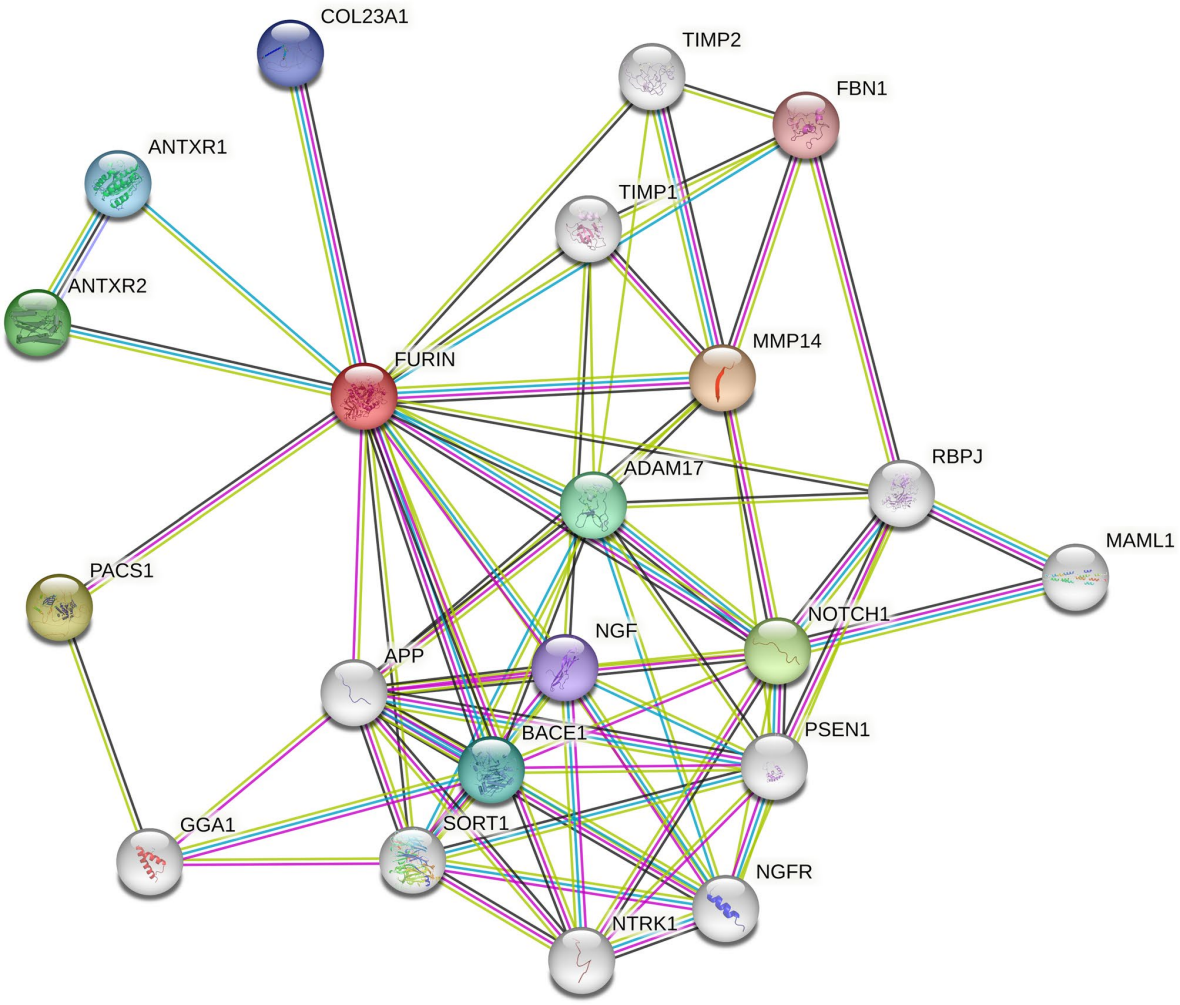
The STRING protein–protein interaction (PPI) analysis queried with FURIN (using STRING V.11.5). Each node represents proteins. Filled nodes represent some 3D structure that is known or predicted. Empty nodes represent proteins of unknown 3D structure, coloured nodes represent query proteins and the first shell of interactors, while white nodes represent the second shell of interactors. Edges represent protein–protein associations. Coloured lines between proteins show different types of interaction evidence. Known interactions (sky-blue line: from curated databases; pink line: experimentally determined). Predicted Interactions (blue line: co-occurrence; green line: gene neighbourhood; red line: gene fusion). Others (black line: co-expression; yellow line: text mining; purple line: protein homology).

The string network shows 21 nodes and 73 edges representing proteins and protein–protein interactions, respectively. The network shows the PPI enrichment (p-value = 1.55e−13), Suggesting that the nodes are not random and that the observed number of edges is significant (Fig. 2 and Supp. Table S4a). We also look for Functional enrichments in our network. The network's biological process shows it has a significant role in Inflammatory response (FDR = 0.0094),Inflammatory response to antigenic stimulus (FDR = 0.0268), and Human Phenotype shows Pulmonary arterial hypertension (FDR = 0.0309) (Supp. Table S4b).

### Analysis of Furin gene variant association with COVID-19 CFR

**Figure 3:**
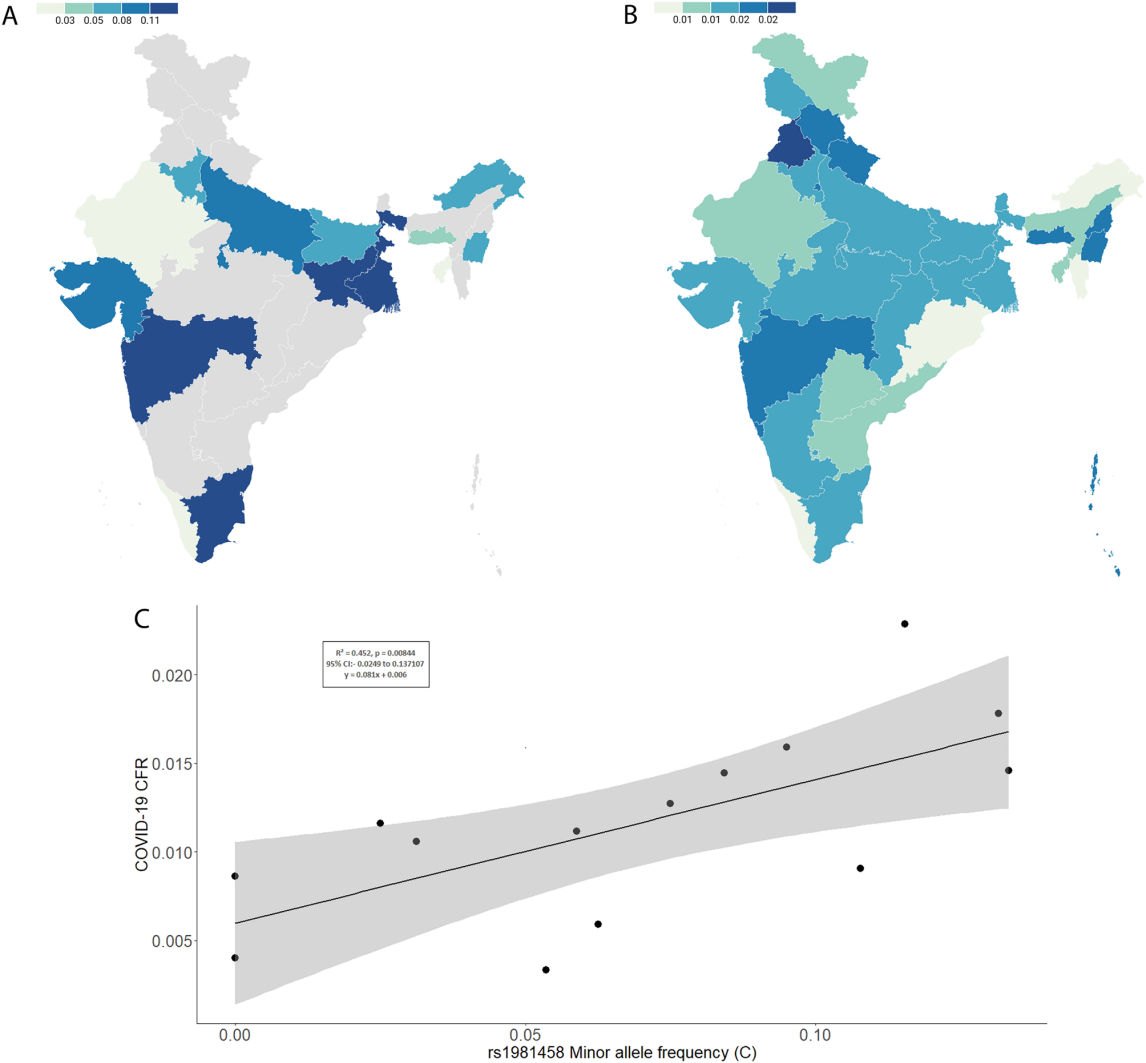
(**A**) and (**B**) display the allele frequency distribution of rs1981458 across Indian populations in a frequency map and the corresponding COVID-19 Case Fatality Rate (CFR) as of August 30th, 2021. The grey colour indicates a lack of data. These visualizations were created using Datawrapper (https://www.datawrapper.de/). (**C**) The linear regression graph depicts the association between the rs1981458 allele frequency of the FURIN gene with COVID-19 CFR.

**Figure 4:**
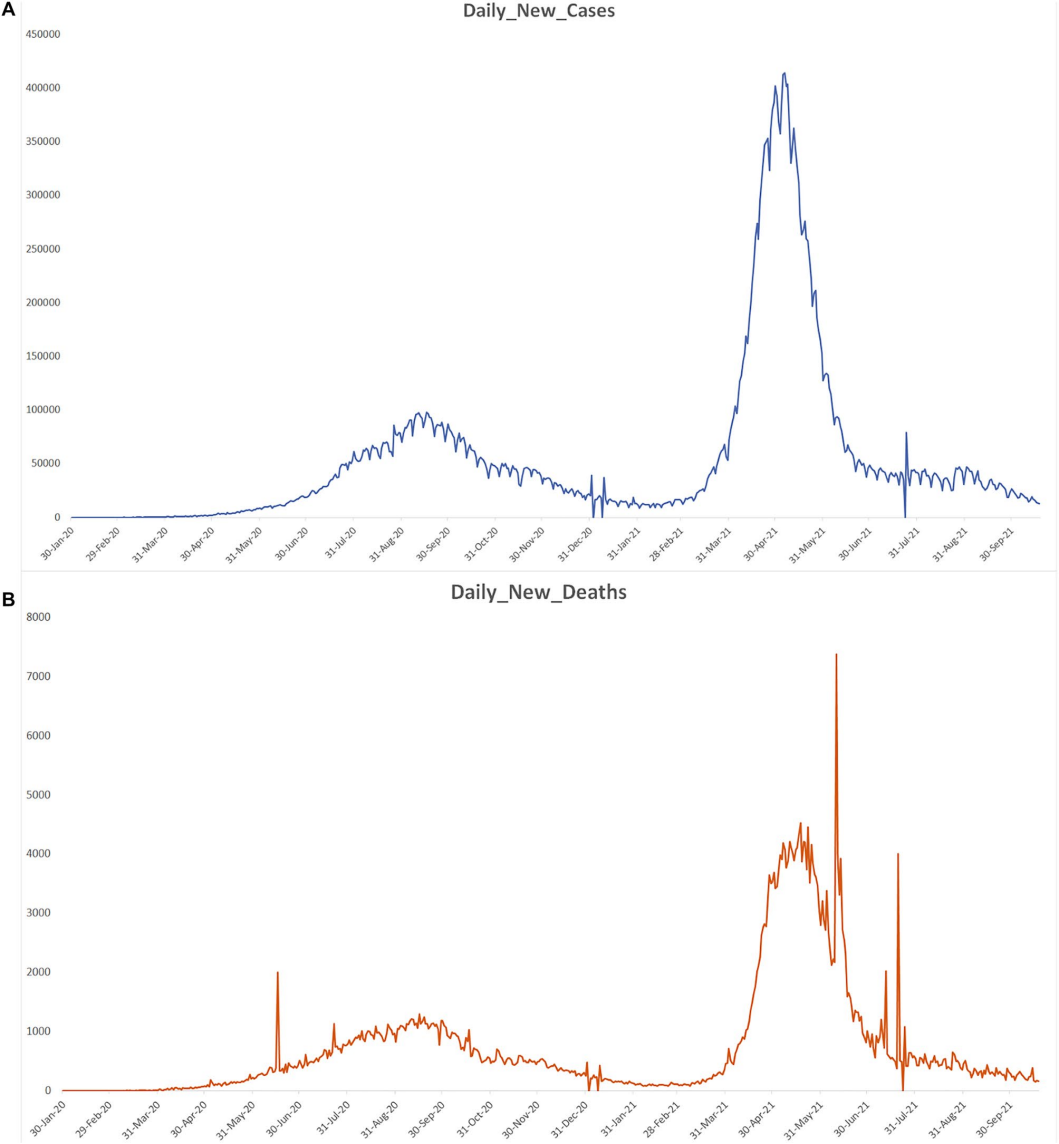
The figure depicts the timeline of Daily new cases and deaths during 1st and 2nd wave.

The Linear Regression Analysis showed a significant positive correlation for rs1981458 SNP between allele frequency and case fatality rate (p < 0.05). Higher CFR was observed where the allele frequency is high and vice versa (Fig. 3A–C and supplementary Table S5). The goodness of fit (R^2^) explained 45.2% of the variation, suggesting a large effect size of this allele in the Indian population. Among the diverse population studied across 14 Indian states, 10 populations—namely Gujarat, Haryana, Kerala, Manipur, Meghalaya, Rajasthan, Tamil Nadu, Tripura, Uttar Pradesh, and West Bengal—demonstrated associations falling within the 95% CI interval. Since this is an active pandemic with changing numbers of infected and dead patients (Fig. 4), we confirmed our findings at 5 different timelines of the first and second waves with epidemiological data available on COVID-19. We found that our results are consistent without any significant difference (Table 1).

**Table 1:** The outcome of the test was performed for statistical significance during different timelines of 1st and 2nd waves of the pandemic**.**

Observation	Linear regression analysis parameters			
rs1981458	R^2^	Effect size (β)	Std. error (S_e_)	p-value
14 Sep_2020_CFR	0.3462	0.588	0.006905	0.0269
01 Jan_2021_CFR	0.4275	0.654	0.004706	0.0112
14 Mar_2021_CFR	0.4520	0.672	0.004146	0.0084
07 May_2021_CFR	0.4360	0.660	0.002925	0.0102
15 Jul_2021_CFR	0.3599	0.600	0.003562	0.0233

### Spatial distribution and diversity of rs1981458

Our spatial analysis revealed that the frequency of this SNP (rs1981458) in India varied between 0 and 13%, the lowest being in Kerala and Rajasthan, i.e., 0%, while the highest in Maharashtra and Tamil Nadu. Northeastern states (Arunachal Pradesh, Meghalaya, Manipur, and Tripura) exhibit low frequency, i.e., 2–7% (Supp. Table S1a). We also looked for the worldwide distribution of rs1981458 from 1000 genome data. We found that the frequency of rs1981458 frequency was highest in Africans (0.2617) while lowest in East Asians (0. 0050) (Supp. Table S6 and Supp. Fig. S2). Similarly, the highest Heterozygosity (HET) and Nucleotide diversity (PI) were found in Africa, while East Asia shows the lowest Heterozygosity and Nucleotide diversity. χ^2^ test of Hardy–Weinberg equilibrium (HWE), Tajima's' D statistic (Tajima.D), Fu & Li's' F* statistic (Fu.Li.F) and Fu & Li's' D* statistic (Fu.Li.D) score suggests that this SNP indicates likely to be influenced by selection and population history (Supp. Table S7).

### QTL analysis of rs1981458

**Figure 5:**
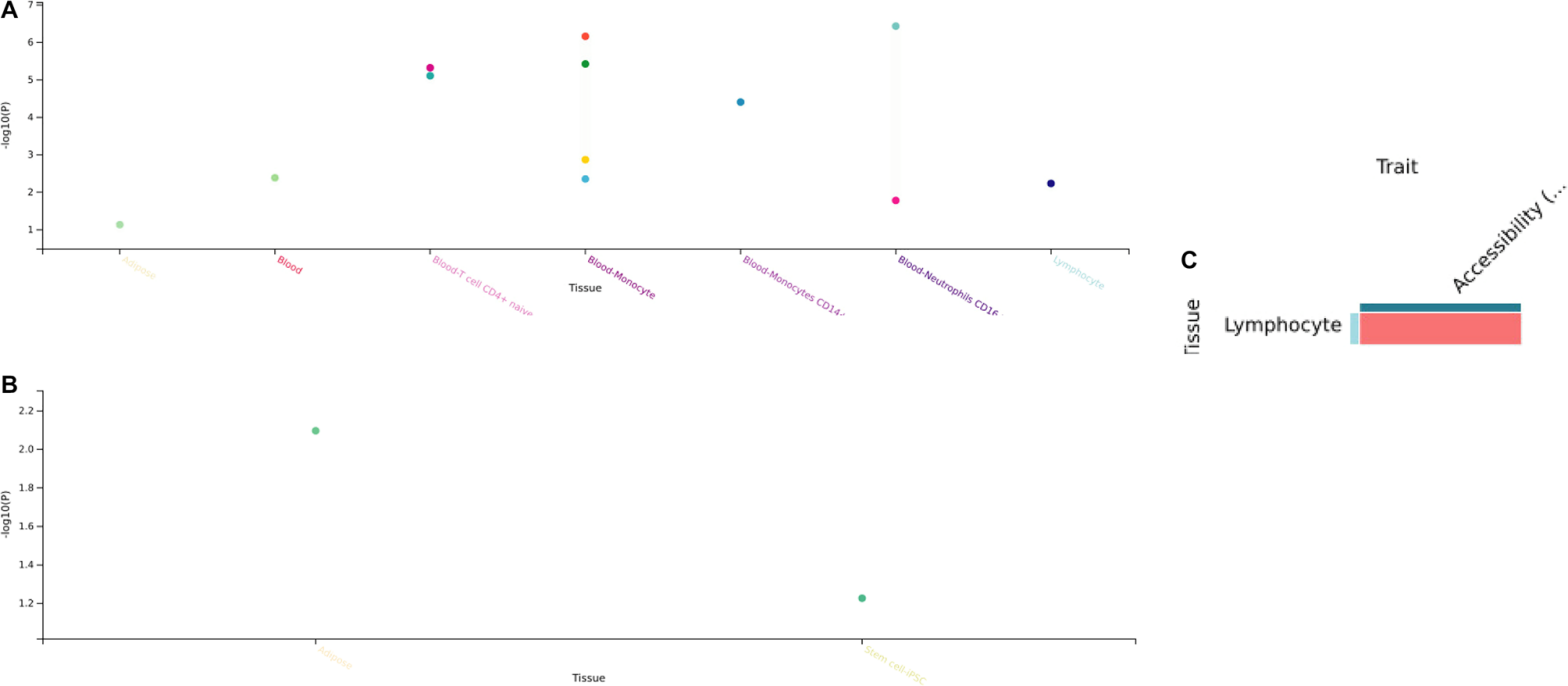
(**a**–**c**) represent eQTL, sQTL and caQTL plots, respectively, for rs1981458-FURIN association among Tissue(s).

We found 12 significant eQTL (p > 0.05) for rs1981458-FURIN across seven tissues, mainly in Immune cells. Most significant associations were found in Blood-Neutrophils CD16 + and Blood monocytes (Fig. 5a and Supp. Table S8a). sQTL (splicing quantitative trait locus) for rs1981458-FURIN shows a significant result in adipose tissue (Fig. 5b and Supp. Table S8b). caQTL (chromatin accessibility quantitative trait locus) shows a significant association in lymphocytes (Fig. 5c and Supp. Table S8c).

### Functional annotation of rs1981458

Annotation of the rs1981458 shows that it is located on the Furin gene on chromosome 15:90873375 (GRCh38) as Ref: T and Alt: C alleles. Transcript Annotation of rs1981458 shows it as a 5' UTR intron variant. Roadmap and Epimap epigenomics data show this SNP mainly to be upregulating. The highest number of linked SNPs was found in Europe (Supp. Table S9a). Genome-scale Pathogenicity Score showed this SNP as likely to be pathogenic (Supp. Table S9b). Genome-scale Oncogenicity Score shows rs1981458 as the likely cancer driver (Supp. Table S9c).

RegulomeDb shows 41 peaks, a score of 0.69529 and ranks as 3a for this variant, which indicates TF binding + any motif + DNase peak both suggests that this variant is likely to have a regulatory role. Its chromatin state showed 127 results, of which 44 are in Active state, 14 are strong transcription, and 69 are predicted to be Enhancers. Experiments based on FAIRE-seq and DNase-seq show that this variant is accessible in 16 tissues, including the lungs.

**Figure 6:**
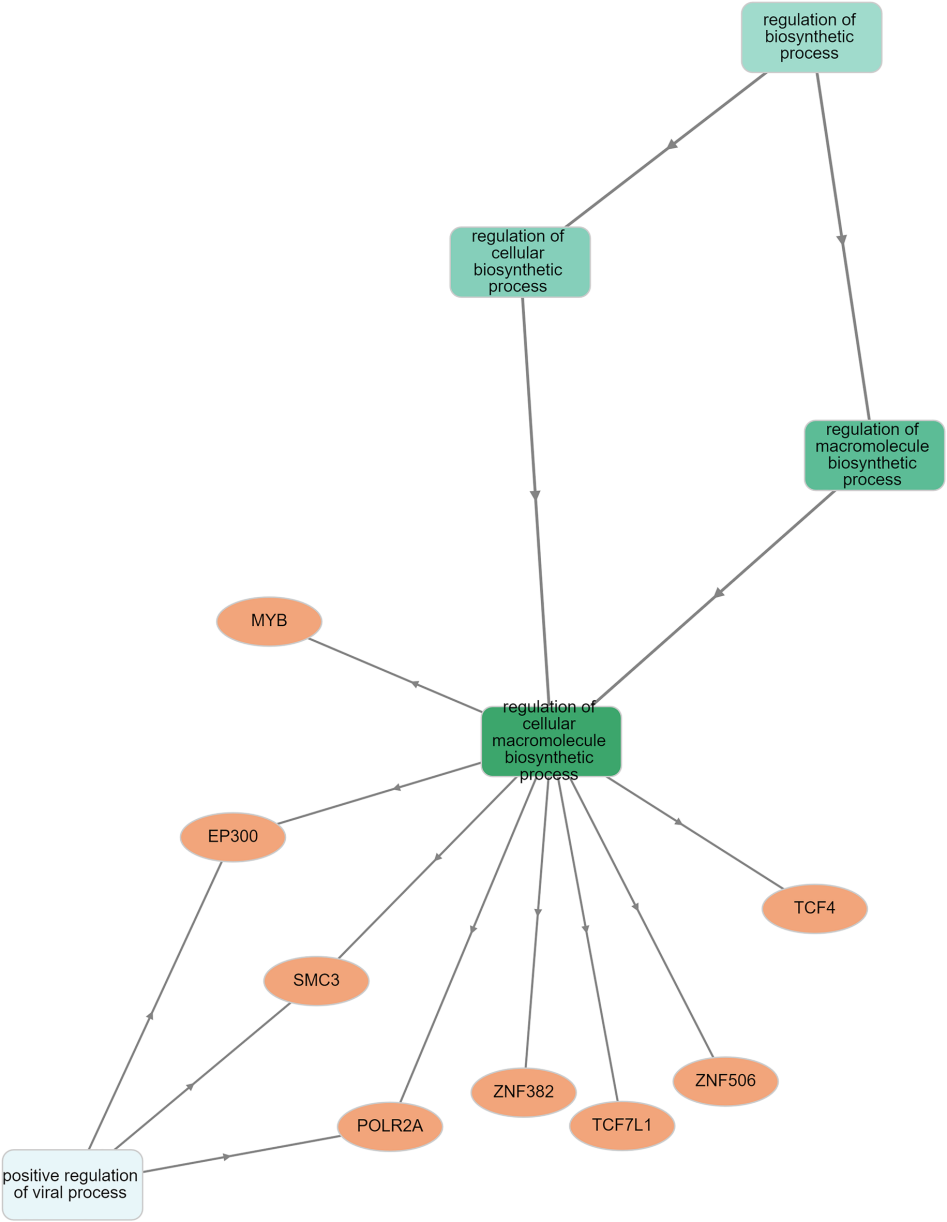
The figure represents gene ontology in terms of biological process based on the Transcription factor binding evidence data from the Vanno Portal.

Overlapped transcript factor binding peaks measured by ChIP-seq from VannoPortal and RegulomeDb show the binding of 9 and 24 transcription factors, respectively (Supp. Table S10a,b). We Analyse these gene/protein signatures in relation to GO terms to know their biological function for these two sets of datasets. We found it plays a role in the positive regulation of viral processes (q-value threshold < 0.05 and p-value threshold 8.46e−6) for VannoPortal data. Similarly, we found significant positive regulation of the viral process, regulation of viral transcription, and positive regulation by the host of viral transcription (q-value threshold < 0.05 and p-value threshold 3.87e−4) for RegulomeDb data (Fig. 6, Supp. Fig. S3, Supp. Table S11a,b).

### rs1981458 pathway analysis

**Figure 7:**
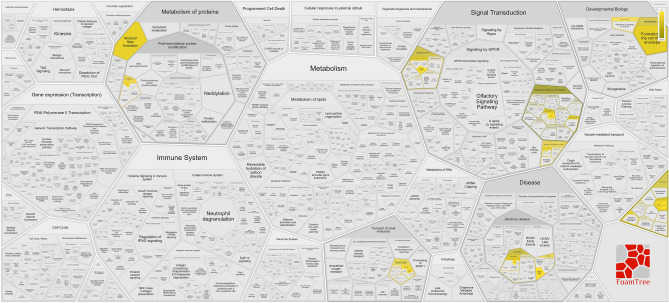
Shows the Reactome pathway associated with rs1981458 in the biological processes in the Voronoi diagram. The colour bar shows how the colour intensity represents the p-value of the statistical test for overrepresentation.

Pathway analysis for rs1981458 using SNPNEXUS shows 37 significantly enriched pathways (p < 0.05) ranging from signal transduction, Extracellular matrix organisation, Metabolism of proteins, Transport of small molecules, and Developmental Biology to Infectious disease. The most significant association for this SNP was found for the Synthesis and processing of ENV and VPU (p-value = 0.000093) (Fig. 7 and Supp. Table S12).

### Phylogeographic structure of Furin gene

**Figure 8:**
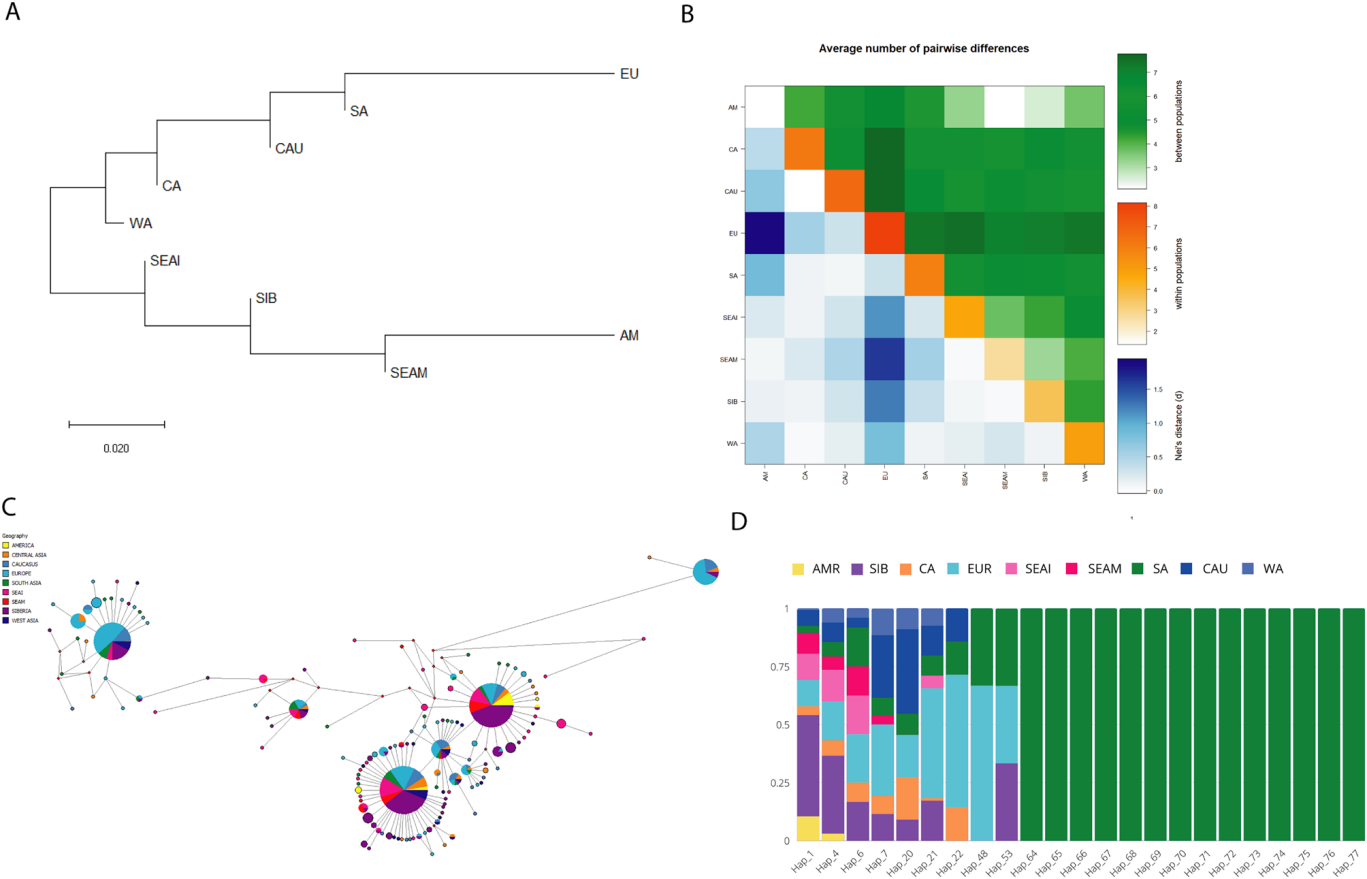
(**A**) A Neighbour-Joining (NJ) tree constructed using Fst distance demonstrates the phylogenetic relationship shared by the FURIN gene in the population under study. (**B**) Matrix displaying average paired variation for the FURIN gene, with within-population variation (orange) along the diagonal, Nei's distance between populations (blue) in the bottom triangle, and variance between populations (green) in the top triangle. The computed value for various variables is directly correlated with the gradient of colour. (**C**) The median-joining (MJ) network of 142 haplotypes belonging to the Furin gene. Circle sizes are proportional to the number of samples with that haplotype. Each colour corresponds to a geographic location. (**D**) The stacked bar plot depicts the 47 FURIN gene haplotypes discovered in South Asian populations. Differently coloured bars show the frequency and sharing for each haplotype in South Asia and other geographical areas.

The Neighbour Joining tree based on Fst distances clustered South Asians with West Eurasian populations (Fig. 8a), suggesting a closer genetic affinity of South Asians with West Eurasians for the Furin gene.Similarly, the Average Pairwise Differences findings indicate smaller genetic distance and Average Pairwise Differences within East and West Eurasian groups, respectively, but greater genetic distance and Average Pairwise Differences between East and West Eurasian populations groups. In comparison to American; Southeast Asian, Mainland, and Siberian populations, Europeans had high Average Pairwise Differences within the population (Fig. 8b). According to median-joining network analysis of the Furin gene, there is a total of 137 haplotypes among the populations studied, with four major haplotypes (Hap_1, Hap_4, Hap_16 and Hap_21). Hap_1 and Hap_4 are more common in Siberia, while Hap_16 and Hap_31 are more common in European populations (Fig. 8c and Supp. Table S2c). South Asia populations carry 23 haplotypes, among which 9 are shared (Hap_1, Hap_4, Hap_6, Hap_7 Hap_20, Hap_21, Hap_22, Hap_48, and Hap_53) with other continental populations, while the rest are unique to South Asia. Among the shared haplotypes, seven are shared majorly with the West Eurasian populations, whereas haplotypes Hap_1 and Hap_4 are shared majorly with the East Eurasian populations. (Fig. 8d and Supp. Table S2c). Overall, haplotype sharing and Fst analysis are consistent with the West Eurasian affiliation of most South Asian FURIN gene haplotypes.

## Discussion

A distinguishing characteristic of SARS-CoV-2 is its embodiment of a polybasic site cleaved by Furin^35,36^. The furin protease recognises the canonical peptide sequence RX[R/K]R↓X, where a down arrow and X indicate the cleavage site is any amino acid^15,37^. In SARS-CoV-2, the recognition site is formed by the incorporated 12 codon nucleotide sequence CCT CGG CGG GCA, which corresponds to the amino acid sequence PRRA. This sequence is upstream of arginine and serine, forming the spike protein's S1/S2 cleavage site (PRRAR↓S)^38^. Although such sites are a commonnaturally occurring feature of other viruses within the Subfamily Orthocoronavirinae^16^, it appears in few other viruses from the Beta-CoV genus^39^ It is unique among members of its subgenus for such a site^36^. It was suggested that acquiring the furin-cleavage site in the SARS-CoV-2 S protein was essential for zoonotic transfer to humans^9^. Moreover, it appears to be an important element in enhancing its virulence^40^.

Given the importance of the Furin gene in COVID-19 pathology, the Furin gene expression may be directly linked to varying disease susceptibility. Therefore, to analyse the expression profile of Furin in various tissues, we performed the expression analysis from the GTEx database and found that Furin is expressed highly in the Liver, Lungs, Thyroid, and whole blood (Fig. 1). Since the Lungs are the primary organ affected by COVID-19 infection, the high expression of Furin in the lungs highlights its importance in COVID19. A Recent study which aims to investigate the levels of mRNA expression of Furin in the peripheral blood of COVID-19 patients with different disease severity to understand if there is any correlation the disease severity reported a Statistically significant severe positive correlation in mRNA expression levels of Furin and, therefore, may affect COVID-19 susceptibility and severity^41^.

We also looked for Bulk RNA Expression of the Furin Gene in various Immune cell types. It shows differential expression of Furin in these immune cells. High Furin expression was found in NK cells, Monocytes (classical), Th17, and TREG cells. These cell types are involved in various immune functions, including antiviral responses. Higher Furin levels in these cells could imply an Increase potential for viral entry, making these cells more susceptible to infection.

In comparison, Naive B cells and naïve CD4 T cells showed the least Furin expression of the furin gene. These cells are crucial for antibody production and adaptive immune response development. Lower Furin levels might indicate delayed or less efficient processing of immune molecules, possibly impacting subsequent immune activation and antibody production. The differential expression of Furin in these immune cells suggests potential involvement in modulating immune responses against COVID-19.

Proteins rarely work alone and interact with other proteins through protein–protein interaction, which plays a crucial role in predicting the protein function of the target protein. Therefore, we also analyse for the FURIN protein–protein interactions network and its role in biological processes. We found a significant role of these interactions in Inflammatory response and Pulmonary arterial hypertension. The inflammatory response is one of the crucial hallmarks of COVID-19 (Supp. Table S4b). Furthermore, some recent studies revealed that patients who died of COVID-19 exhibited thickened pulmonary vascular walls, a crucial hallmark of pulmonary arterial hypertension (PAH)^42^. Therefore, we hypothesise the role of Furin protein–protein interactions in the PAH via a typical inflammation mechanism.

Furin plays a vital role in the SARS-CoV-2 entry into the host cell. Therefore, variations in this gene linked with elevated Furin gene expression could be epidemiologically associated with disease severity outcomes among populations. However, so far, there has not been any study on Furin gene variants among Indian populations. Therefore, we explored the role of the Furin gene variant in the disease severity among the Indian population; for this, we calculated statewide COVID-19 CFR and frequency of the Furin variants and performed the linear regression analysis to understand the correlation of allelic frequency with respect to the COVID-19 CFR among Indian populations. Interestingly, we found a significant positive association for rs1981458 with COVID-19 CFR (Table 1). Therefore, a population with a high rs1981458 allele frequency may account for severe consequences of SARS CoV-2 infection (Fig. 3A–C and Supplementary Table S5), due to the dynamic nature of the pandemic and the fluctuating numbers of infected and deceased people. Therefore, we used epidemiological data on COVID-19 to confirm our observations at 5 different timelines of first- and second-wave timeframes (Fig. 4). We found no significant differences between our observations at these varied timelines (Table 1). After the second wave, there was a massive vaccination drive; thus, later observation was not analysed as it will not represent a true picture of COVID-19 susceptibility.

The spatial distribution of rs1981458 shows the highest frequency of this genetic variant in Maharashtra and Tamil Nadu states while the lowest in Kerala and Rajasthan. This is in accordance with the ground zero data observed, which clearly shows that Maharashtra and Tamil Nadu are most affected and, therefore, have higher CFR compared to other states, while Kerala and Rajasthan are among the states that are least affected and show lower CFR (Fig. 3A,B and supplementary Table 5). We also looked for the worldwide distribution of rs1981458 from 1000 genome data (Supp. Fig. 2 and Table 2) and found that rs1981458 frequency was highest in Africans (0. 2617) while lowest in East Asians (0. 0050) populations. Interestingly, East Asians also showed the least severity of COVID-19. A recent study has suggested the adaptation of many genes that engage with coronaviruses, including the SARS-CoV-2, which began 25,000 years ago for coronaviruses or a related virus outbreak in East Asia^43^. Therefore, the low frequency of this allele in the East Asian population may be attributed to such evolutionary selection. These findings are consistent with epidemiological data available on COVID-19, which shows that people of East Asian ethnic background are the least affected. India also shows similar observations where North-East Indians, composed mainly of Tibbato-Burmese speakers harbouring major East Asian-related Ancestry, are least affected (Fig. 3b).

**Table 2:** Frequencyof rs1981458 from 1000 Genomes database.

Region	Rs1981458	
	Ref. allele (T)	Alt. allele (C)
African	0.7383	0.2617
East Asian	0.9950	0.0050
European	0.8966	0.1034
South Asian	0.909	0.091
American	0.934	0.066

To understand the functional relevance of rs1981458, we undertook several analyses to characterise its functional and regulatory role in the biological system. eQTL analysis showed various significant associations, mainly in Immune cells (Fig. 5a), thus highlighting the role of this SNP in host immunity. Furthermore, most eQTL were present on the APC cells and Lymphocytes, suggesting this SNP plays a crucial role in virus presentation and processing for recognition by lymphocytes such as T cells. caQTL also showed a significant association with lymphocytes, suggesting its accessibility in lymphocytes (Fig. 5c). sQTL shows a significant association in adipose tissue (Fig. 5c). Cholesterol and substrate presentation are known to control the expression of Furin. Furin moves to GM1 lipid rafts when the level of cholesterol is high. Conversely, when cholesterol is low, Furin traffic to the disordered region^44^; therefore, it may contribute to cholesterol and age-dependent priming of SARS-CoV.

This SNP is annotated in the 5'UTR region, which is vital for the regulation of translation. The epigenomics data from Roadmap and Epimap indicate that this SNP is upregulating. RegulomeDB data suggest this SNP has a regulatory role with enhancer function and is in an accessible state with a strong transcription function (https://regulomedb.org/regulome-search/). According to the Pathogenicity Score, this SNP was likely pathogenic (Supp. Table S9b). The Oncogenicity Score signals that rs1981458 is probably a cancer driver (Supp. Table S9c) as this SNP is in the vicinity of the FES; an oncogene, therefore, may have a role in cancer.

We analyse transcript factor binding peaks measured by ChIP-seq from VannoPortal and RegulomeDb for their biological function for these two datasets. We found it plays a significant role in the positive regulation of the viral process, regulation of viral transcription, and positive regulation by the host of viral transcription (Fig. 6, Supp. Fig. S3, and Supp. Table S11a,b). We also looked for pathway enrichment of rs1981458; the most significant association for this SNP was found for the Synthesis and processing of ENV and VPU) (Fig. 7 and Supp. Table S12). Env and Vpu, two viral membrane proteins transcribed by the same mRNA, are translated on the rough ER. Every virion component must travel from its point of synthesis to its assembly site on the plasma membrane. Env is an integral membrane protein. It is inserted co-translationally into ER membranes and then travels through the cellular secretory pathway, where it is glycosylated, assembled into trimeric complexes, and processed into the gp41 and gp120 subunits by the cellular protease furin^45^, therefore validating its role in viral processing from the previous observation.

The genetic structure of the Furin gene among South Asian populations is largely unknown, and knowing this can help understand the role of the Furin gene in host disease susceptibility in South Asia with respect to the global population. Therefore, mainly relying on the haplotype-based approach, we have analysed the whole genome data of the Furin gene to compare South Asians to other world populations. The results show the South Asians affinity towards West Eurasia populations (Fig. 8A). Therefore, the host susceptibility of SARS-CoV-2 for the Furin gene among South Asians is most likely expected to be like West Eurasians rather than East Eurasians. In contrast, our previous study on the ACE2 and TMPRSS2 genes has shown thecloser genetic affinity of South Asian haplotypes with the East Eurasians and West Eurasians, respectively^11,12^. As a result, it is worth proposing that the South Asian population's susceptibility to SARS-CoV-2 will be more inclined to the West Eurasian population.

The implications of these findings in the broader context of COVID-19 research are vast and multifaceted. Utilising the Insights from genetic markers like rs1981458 and the genetic structure of Furin can aid in identifying communities at higher risk and stratification for COVID-19 susceptibility. Healthcare systems can leverage this information to better preparedness, prioritise resources, and tailored interventions in regions with higher genetic vulnerability to severe COVID-19 outcomes. Understanding the regulatory potential of rs1981458 provides valuable insights into the role of Furin in disease pathology, opening avenues for drug development and allowing researchers to focus on specific pathways or molecular mechanisms, such as immune response modulation or viral assembly and processing, for more effective treatment strategies. In essence, these findings add depth to our understanding of COVID-19 susceptibility and hold promise for personalised healthcare, targeted interventions, and more effective global responses to current and future infectious diseases.

## Conclusion

In conclusion, for the first time, we have shown the role of the Furin gene variant in COVID-19 severity among Indian populations. We found a significant association between the SNP rs1981458 allele and COVID-19 CFR. This study also outlines the positive regulatory potential of rs1981458 in COVID-19. We also analysed the genetic architecture of the Furin gene and found that South Asians and West Eurasian groups have a closer genetic affinity. Therefore, it is worth proposing that the Furin gene COVID-19 susceptibility of South Asians will be more similar to the West Eurasian population.However; we emphasise the need for cautious interpretation due to the study's limitations. We believe this insight may well be utilised as a genetic biomarker to identify vulnerable populations, which might be highly helpful for developing policies and allocating resources more effectively during an epidemic.

## Limitations and future research

We caution that SNP rs1981458 is only one of many variables affecting the severity of COVID-19, e.g., an individual's age, sex, pre-existing comorbidity, the strain of the virus, population density, social distancing, access to healthcare facilities lockdown, etc., can all significantly affect the infection rate and CFR and lack of data on these factors remain the key limitation of the current study. Furthermore, we duly note the observational nature of our research and the absence of a comparative cohort comprising non-Indian participants. Future studies integrating diverse cohorts and encompassing other multifaceted factors influencing COVID-19 severity are important to deepen our understanding of this complex landscape. Therefore, future research should incorporate comparative cohorts from various regions exploring a wider range of genetic, environmental, lifestyle and socio-economic influences to understand global variations in genetic susceptibility to COVID-19 severity.

### Supplementary Information

Supplementary Tables.

Supplementary Figures.

## Data Availability

All datasets generated for this study are included in the article/Supplementary Material.
