# Author Correction: Novel genetic association of the Furin gene polymorphism rs1981458 with COVID-19 severity among Indian populations

**DOI:** 10.1038/s41598-024-60182-8

**Published:** 2024-04-25

**Authors:** Rudra Kumar Pandey, Anshika Srivastava, Rahul Kumar Mishra, Prajjval Pratap Singh, Gyaneshwer Chaubey

**Affiliations:** Cytogenetics Laboratory, Department of Zoology, Banaras Hindu University, Varanasi, 221005 India

Correction to: *Scientific Reports* 10.1038/s41598-024-54607-7, published online 03 April 2024

The original version of this Article contained errors in the Material and Methods section and in the legend of Figure 3 (A, B, C).

In the Material and Methods section, under the subheading ‘Insilco analysis’,

“To analyse the TMPRSS2 expression in various human tissues, the GTEx portal database (http://www.gtexportal.org/home/) was used.”

now reads:

“To analyse the FURIN expression in various human tissues, the GTEx portal database (http://www.gtexportal.org/home/) was used.”

In the legend of Figure 3,

“(**A**) and (**B**) display the allele frequency distribution of rs2070788 across Indian populations in a frequency map and the corresponding COVID-19 Case Fatality Rate (CFR) as of August 30th, 2021.”

now reads:

“(**A**) and (**B**) display the allele frequency distribution of rs1981458 across Indian populations in a frequency map and the corresponding COVID-19 Case Fatality Rate (CFR) as of August 30th, 2021.”

“The linear regression graph depicts the association between the rs1981458 allele frequency of the TMPRSS2 gene with COVID-19 CFR.”

now reads:

“The linear regression graph depicts the association between the rs1981458 allele frequency of the FURIN gene with COVID-19 CFR.”

The original Article has been corrected.

