# Supplementary material for: Novel genetic association of the Furin gene polymorphism rs1981458 with COVID-19 severity among Indian populations
Source: Sci Rep. 2024 Apr 3;14:7822. doi: 10.1038/s41598-024-54607-7 (PMC10991378; doi:10.1038/s41598-024-54607-7)
Supplement: Supplementary file 2 — Supplementary Figures. [file 41598_2024_54607_MOESM2_ESM.docx]

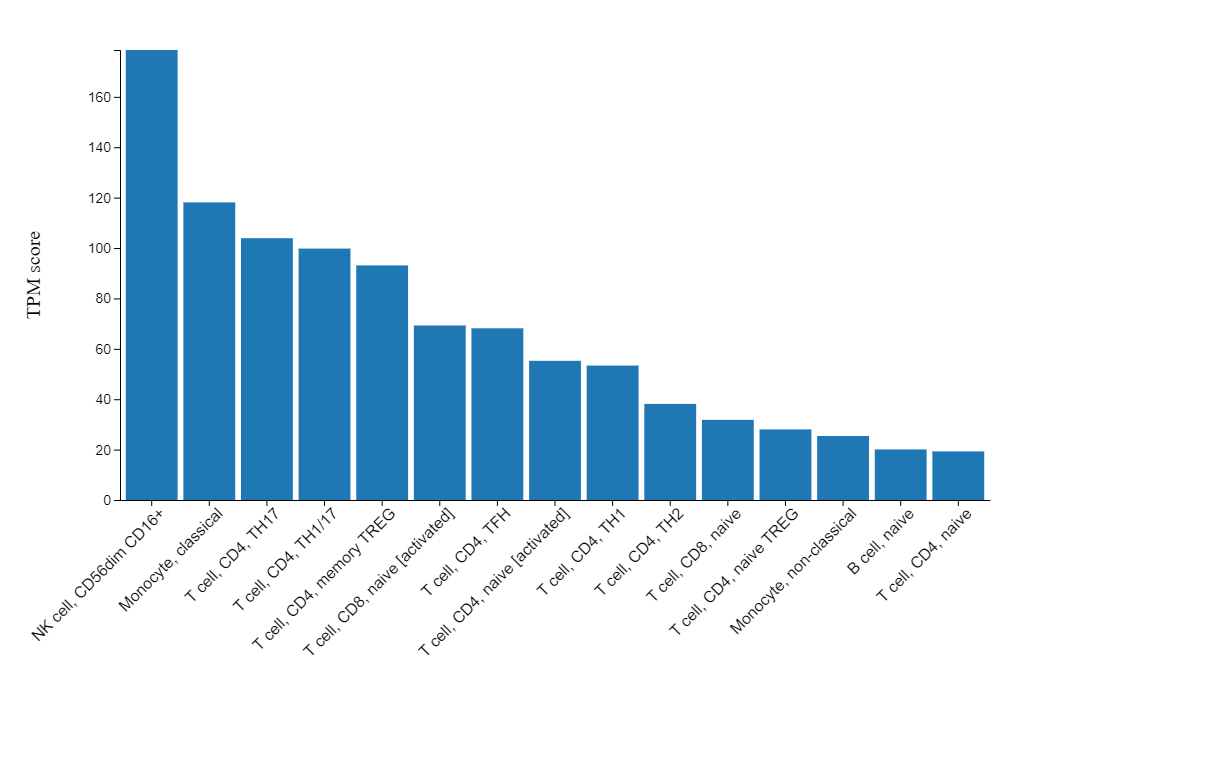


**Supp. Figure 1.** The figure depicts the expression of the Furin gene in various immune cells based on the ascending order of TPM score (Transcription per million value).


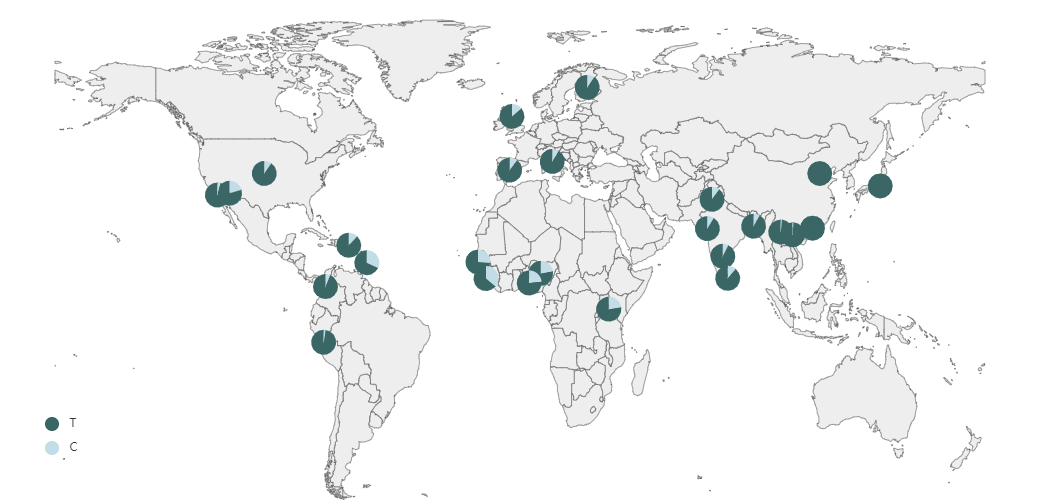


**Supp. Figure 2. The frequency** distribution of rs1981458 in the world population was extracted using 1000 Genome data and plotted on a map using PGG.SNV.


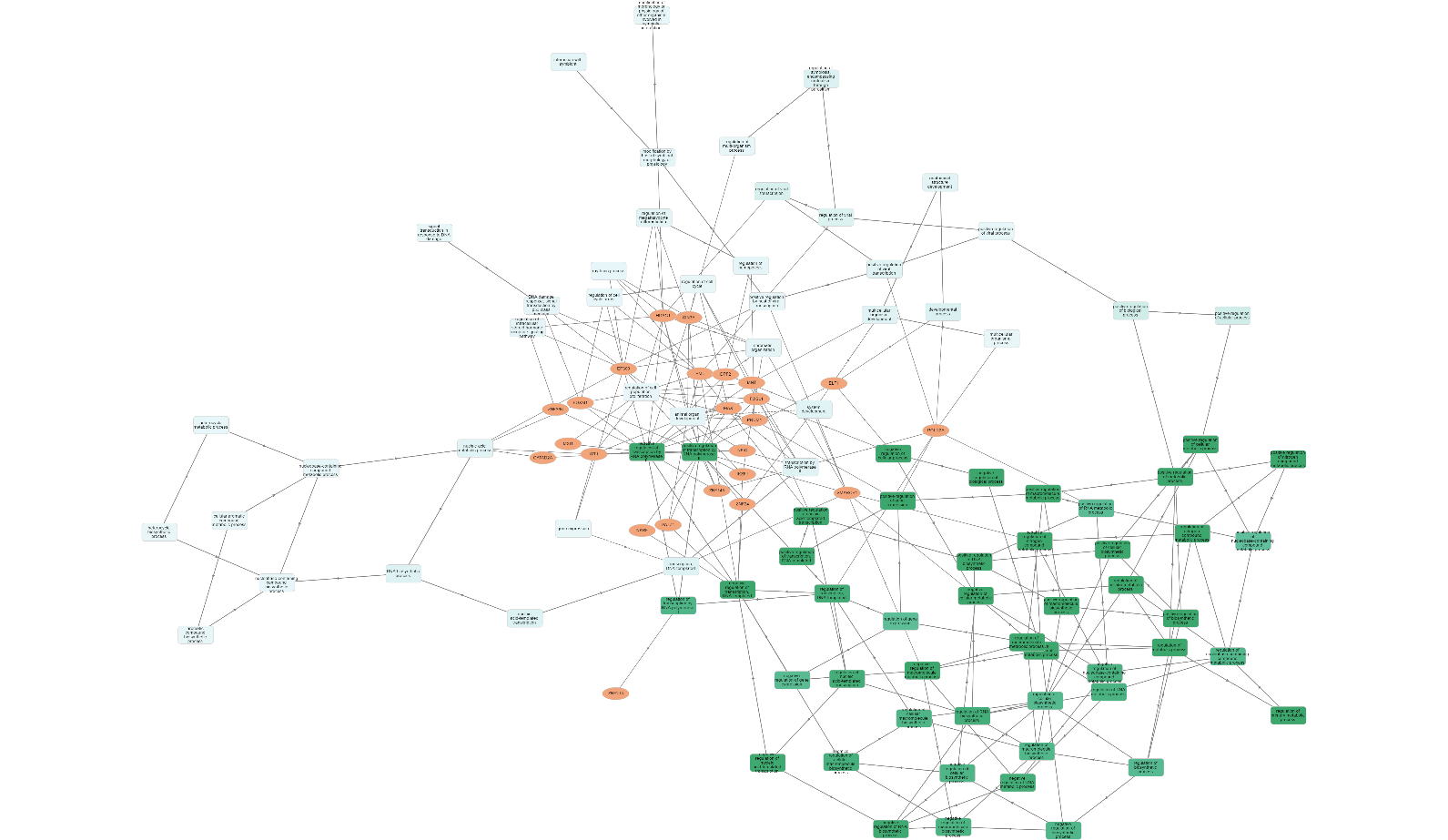


**Supp. Figure. 3.** The figure represents gene ontology in terms of biological processes based on the Transcription factor binding evidence data from the RegulomeDb.
